# Validation of Obesity Status Based on Self-Reported Data among Filipina and Indonesian Female Migrant Domestic Workers in Macao (SAR), China

**DOI:** 10.3390/ijerph17165927

**Published:** 2020-08-15

**Authors:** Lei Huang, Wen Chen, Andre M. N. Renzaho, Brian J. Hall

**Affiliations:** 1 Jockey Club School of Public Health and Primary Care, The Chinese University of Hong Kong, Hong Kong 999077, China; rebeccaleihuang@gmail.com; 2 Global and Community Mental Health Research Group, Department of Psychology, Faculty of Social Sciences, University of Macau, Macau 999078, China; 3 Department of Medical Statistics, School of Public Health, Sun Yat-sen University, Guangzhou 510000, China; chenw43@mail.sysu.edu.cn; 4 Sun Yat-sen Centre for Migrant Health Policy, Sun Yat-sen University, Guangzhou 510000, China; 5 School of Social Sciences and Translational Health Research Institute, Western Sydney University, Penrith 2750, Australia; Andre.Renzaho@westernsydney.edu.au; 6 Maternal, Child and Adolescent Health Program, Burnet Institute, Melbourne 3004, Australia; 7 Department of Health Behavior and Society, Johns Hopkins Bloomberg School of Public Health, Baltimore, MD 21201, USA

**Keywords:** body mass index, height, weight, self-report, cut-off, migrant workers

## Abstract

Background: Migrant domestic workers are at high risk of overweight and obesity. It is crucial to assess the prevalence of obesity among this migrant population, for surveillance and intervention. Self-reported height and weight are commonly used to derive body mass index (BMI) and assess the prevalence of obesity. The accuracy of BMI from self-reported height and weight in migrant populations remains unknown. The aim of this study was to assess the accuracy of BMI from self-reported measures and identify the optimal adjustment to be made to overweight and obesity cut-off points when using self-reported body mass index among migrant workers. Methods: Self-reported and objectively measured height and weight were obtained from 1388 female Filipina domestic workers and 369 female Indonesian domestic workers recruited using respondent-driven sampling between November 2016 and August 2017. Self-reported BMI (based on self-reported height and weight) and measured BMI (based on objectively measured height and weight) were calculated as weight in kilograms divided by the square of height in meters for all participants (kg/m^2^). Results: BMI derived from self-reported height and weight was underestimated for both Filipina (z = −27.5, *p* < 0.001) and Indonesian (z = −9.9, *p* < 0.001) participants. Applying the gold standard of Asian BMI cut-off points to self-reported BMI, the sensitivity in identifying overweight or obesity was 64.4% for Filipina participants and 78.6% for Indonesian participants and the specificity was 97.9% for Filipina participants and 93.8% for Indonesian participants for overweight or obesity. When self-reported measures were used, the receiver operator characteristic (ROC) curves and the corresponding area under the curve (AUC) indicated optimal cut-off points of 22.0 kg/m^2^ and 22.3 kg/m^2^ for Filipina and female Indonesian participants for overweight or obesity. Conclusions: Although BMI derived from self-reported height and weight allows for quick and low-cost obesity screening, a considerable underestimation of overweight or obesity prevalence was observed in Filipina and female Indonesian migrant domestic workers in Macao (Special Administrative Region, SAR), China. With the best compromise between sensitivity and specificity, the new cut-off points can be used in future studies to identify overweight or obesity in these two populations using self-reported height and weight.

## 1. Introduction

Overweight and obesity have become pandemic [1,2]. The global prevalence for overweight and obesity among adults reached 39% and 13%, respectively, in 2016, indicating that more than 1.9 billion adults were overweight or obese throughout the world [3]. Over the past decades, the prevalence of overweight and obesity among adults has been rising in both low-income and high-income countries [4]. While the rate of increase has slowed down in most high-income countries, it remains high in low- and middle-income countries, which implies a shift in overweight and obesity risk by region [4,5]. Although overweight and obesity have long been more prevalent in high-income countries, the latest estimates show that nearly two-thirds of the world’s obese individuals live in low- and middle-income countries [4]. As a growing problem in many low- and middle-income countries, overweight and obesity require public health action.

Overweight and obesity are important risk factors for cardiovascular diseases, diabetes, and cancer [6,7,8]. The disease burden related to overweight and obesity has been increasing in the past decades [9]. The global burden of disease reveals that a high body mass index (BMI) accounted for 4.0 million fatalities globally, and over 60% of deaths occurred among obese individuals [9]. Asian transnational migrant populations tend to have a higher prevalence of overweight and obesity compared with native populations [10,11,12,13]. A systematic review on the prevalence of obesity among migrant Asian Indians identified an average of 0.36 higher BMI values for migrant Asian Indian population when compared with other migrants and the native populations [10]. A high prevalence of overweight and obesity was also observed among South Asian migrant workers in the Middle East, with 30% to 66% for overweight and 17% to 80% for obesity [13]. The high prevalence of overweight and obesity also contributes to the high morbidity [14,15] of cardiovascular diseases in Asian transnational migrant populations. However, these studies were all conducted among migrant populations from South Asia, particularly from India. The prevalence of overweight and obesity, as well as the associated cardiovascular risk, remains largely under investigated among transnational migrant populations from Southeast Asia, which is another subregion of Asia with a long history of transnational migration [16]. According to the International Labour Migration Statistics (ILMS), there are over 18.8 million Southeast Asian migrants worldwide [17]. Assessing the prevalence of overweight and obesity among Southeast Asian migrant populations is a crucial step for future actions on global migrant health.

Overweight and obesity among migrant workers have additional public health impacts in both the host region and the home region. The early onset of obesity is associated with poor physical functioning at an older age [18]. The risk of many cardiovascular diseases also increases substantially at an older age [19]. As obesity has long-term health consequences throughout the life course, migrant populations, particularly migrant workers, may develop obesity in the host region during the migration process and suffer from the negative consequences of obesity when they return to their home region at an older age. Their home region, therefore, may face great public health challenges in dealing with the increasing burden of disease associated with obesity, overwhelming available public health resources.

Although having a high risk of overweight and obesity, migrant workers have fewer resources to deal with the negative consequences of overweight and obesity owing to time and financial constraints [20]. They tend to have limited access to the healthcare system, and are likely to be excluded from obesity surveillance in both the host region and in their home region [20]. Given a low education level and limited access to health information, they may not be aware of their obesity status and the negative consequences of obesity, which further increases their risk of becoming overweight and obese [21,22]. This emphasizes the need for preventive programs, including screening and preventative interventions for obesity, within this population. Raising awareness of their obesity status is a key first step for obesity intervention programs targeting migrant populations. Therefore, it is necessary to assess the prevalence of overweight and obesity among migrant workers, for surveillance and intervention purposes.

Body mass index (BMI), defined as weight in kilograms divided by the square of height in meters, has been widely used as an indicator for overweight and obesity. In order to better identify high risk populations and facilitate intervention, the World Health Organization [23] proposed a set of general cut-off points for BMI, which classifies people into five groups: underweight (<18.5 kg/m^2^), normal weight (18.5–24.9 kg/m^2^), overweight (25–29.9 kg/m^2^), obese stage I (30–34.9 kg/m^2^), and obese stage II (≥35 kg/m^2^). However, with the same value of BMI, Asian populations tend to have a higher body fat percentage and more abdominal fat compared with Western populations, and both high body fat percentage and more abdominal fat are associated with higher risk for type 2 diabetes and cardiovascular diseases [24]. Therefore, the use of general cut-off points for BMI may underestimate the magnitude of obesity-attributable disease burden in Asian populations, which impairs the allocation of health resources in Asian countries. To address this concern, the WHO Western Pacific Regional Office (WPRO) proposed a different set of BMI for Asian populations, which lowered the cut-off points for overweight and obesity (overweight—23–24.9 kg/m^2^; obese stage I—25–29.9 kg/m^2^; obese stage II—≥30 kg/m^2^) [25]. A study comparing Asians and Caucasians on mortality risk for being overweight found that, for Asians, significant mortality risks started at BMI ≥25.0 kg/m^2^, instead of BMI ≥30.0 kg/m^2^, which further supports the use of WPRO standards for BMI categories in Asian populations [26]. These findings are generalizable to Asian migrant populations who share the same genetic compositions with non-migrant Asian populations, suggesting that WPRO standards for BMI categories should be applied when assessing the prevalence of overweight and obesity among migrant workers.

Self-reported data on height and weight have been commonly collected in epidemiological studies [27,28,29,30]. Compared with objective measures, self-reported measures are easier to obtain and at a lower cost, but are prone to reporting bias. A systematic review of 64 studies on adult various populations suggests that height is generally over-reported and weight is generally under-reported [31]. The reporting discrepancy introduces bias to BMI measurement, which further results in underestimated weight status and obesity prevalence [32]. In addition, the reporting discrepancy also introduces bias into the association between obesity and other health outcomes. Previous studies suggested that the association between obesity and health conditions might be overestimated when self-reported BMI was used [33]. The use of self-reported BMI might also bias the association between BMI and mortality rates [34].

There are two main possible factors contributing to the reporting discrepancy. On the one hand, in certain cultural contexts, the reporting discrepancy may reflect social desirability associated with ideal body image [35]. However, research on the accuracy of self-reported weight, height, and BMI has been conducted primarily among Western populations in high-income countries [31]. The ideal body image and the associated level of body dissatisfaction in these countries may be very different from those in Asian countries [36,37]. The results from the first International Body Project (IBP-I) showed that participants from Southeast Asians preferred higher body weight for females compared with participants in North America and East Asia [37]. For body dissatisfaction, females in Southeast Asia displayed less body dissatisfaction than females in South America and North America [37]. The influence of social desirability on reporting discrepancy may vary for females from Southeast Asia countries, and the accuracy of self-reported measures in Western populations may not be applicable to this population. On the other hand, the accuracy of self-reported measures may provide insights into the degree to which individuals pay attention to their weight status. A huge reporting discrepancy may indicate limited effort or awareness of self-monitoring on weight status [38,39], which implies a gap between self-perceived health status and actual health status. While being vulnerable to poor health outcomes, migrant workers tend to have low level of health awareness and neglect the negative consequences of poor health outcomes [40,41].

Examining the accuracy of self-reported height, weight, and BMI in migrant workers provides evidence on both their self-perceived weight status and actual weight status, which facilitates future public health actions. However, most of the existing evidence on the accuracy of self-reported measures was obtained from non-migrant populations. Only one study included a small group from a rural-to-urban migrant population in Peru, among which a significant proportion of underestimation of BMI was identified [42]. The accuracy of BMI from self-reported height and weight in Southeast Asian migrant populations remains unknown. It is important to assess the accuracy of BMI from self-reported height weight, especially in this population with a high risk of overweight and obesity and low level of health awareness.

The target populations of this study are female Filipina and Indonesian domestic workers in Macao, a Special Administrative Region (SAR) of China. Domestic workers are one of the largest groups of migrant workers in Macao, but their health status is largely understudied [43]. By the end of 2018, there were 28,692 female domestic workers in Macao, of which 15,495 were Filipina, and 3721 were Indonesian [44]. Females are more susceptible to overweight and obesity compared with males [45]. Leaving their home country and working as migrants may further increase their risk of overweight and obesity. Compared with migrant workers in Macao from other Southeast Asian countries, female Filipinas and Indonesians have the highest overweight and obesity prevalence back in their home countries (female Filipina: 24.7% for overweight, 6.1% for obesity; female Indonesian: 28.1% for overweight, 7.8% for obesity) [46]. While they are more likely to bring a high overweight and/or obesity prevalence with them, their anthropometric status may remain undocumented after migrating to Macao. To our knowledge, no study has been conducted among these two populations to examine the accuracy of BMI derived from self-reported height and weight. This study has the following aims:

1. To evaluate the accuracy of BMI and BMI categories based on self-reported height and weight in these two migrant populations.
2. To investigate whether ethnic differences exist between these two populations concerning the discrepancy between self-reported and objective measures.
3. Identify improved overweight and obesity cut-off points for BMI derived from self-reported measures for these two populations.

## 2. Materials and Methods

### 2.1. Participants and Study Design

The Population Research Initiative for Domestic Employees (PRIDE) study is a cohort study that aims to evaluate the overall health status of female migrant domestic workers working and living in Macao (SAR), People’s Republic of China. A total of 1388 female Filipina and 369 female Indonesian domestic workers were included in the PRIDE cohort. The inclusion criteria of the PRIDE cohort are as follows: (1) being 18 years old or above; (2) being a female Filipina or Indonesian domestic worker in Macao (SAR); and (3) holding a valid working visa or a residence ID card of the Macao (SAR).

Respondent-driven sampling [47] was used to recruit eligible participants for the PRIDE cohort from November 2016 to August 2017. The recruitment was conducted independently in female Filipina and Indonesian community. The recruitment process began with participants who have a large network size in each community as “seeds.” The “seeds” were briefed individually by our staff about the purpose and the recruitment process of the study in their native language (Tagalog for Filipina “seeds” and Bahasa Indonesia for Indonesian “seeds”). After the briefing sessions, each of the “seeds” received five uniquely coded coupons to recruit up to five eligible participants in their community. The location and opening hours of the study site were printed on the coupon. When the participants recruited by the “seeds” showed up at the study site, their coupon and working permit (Blue Card) were checked by our research staff to ensure eligibility. Upon completing the baseline study, each of them also received coupons to recruit other eligible participants to the cohort. The recruiter was given 20 MOP (~$2.50 USD) as remuneration for each successful recruit to the cohort. No repeated participation was allowed in the study. The same recruitment procedure was repeated until the required equilibrium and sample size were obtained [48].

The study site was located in a community-based non-governmental organization in Macao (SAR). At least one Tagalog speaker and one Bahasa Indonesia speaker were on duty at the study site and facilitated the data collection. To ensure the quality of data collection, all procedures for data collection were documented, and all research staff were required to follow the documented procedures throughout the data collection period. After providing informed consent, all participants were asked to complete a self-administered survey on tablet devices. The survey was designed to obtain data on demographic characteristics and overall health status, including self-reported height and weight. All the data obtained were saved in a cloud-based system. The data manager was in charge of checking and cleaning the data regularly. Right after the survey, all participants underwent a physical health assessment, in which their height and weight were measured by our trained staff in a private area of the study site. Upon completion, all participants received a brief report on their physical health assessment and 100 MOP (~$13.00 USD) in cash to remunerate their time and effort.

### 2.2. Measures

All participants were asked to report their age and marital status in the survey. The survey was translated into participants’ native language (Tagalog for Filipina participants and Bahasa Indonesia for Indonesian participants). All survey items went through forward and backward translation by bilingual staff. Each item was evaluated using think-aloud cognitive interviewing with a small sample representative of the target population to ensure that the item was relevant, non-offensive, and clear.

#### 2.2.1. Self-Reported Height and Weight

All participants were asked to report their height and weight in the self-administered survey at the study site before they went through the physical health assessment. They were asked to provide their height and weight by answering the question “What is your current height?” and “What is your current weight”, respectively. They could choose to provide their height and weight in either metric or non-metric units, whichever they were more familiar with. All data were later unified to values in metric units (height in cm, weight in kg) for analysis.

#### 2.2.2. Objectively Measured Height and Weight

The height and weight of all participants were measured by our trained staff at the study site. For accuracy, all participants were asked to wear only light indoor clothes and take off their shoes. The height (cm, to the nearest 0.1 cm) was measured by a scale perpendicular to the ground. The weight (kg, to the nearest 0.1 kg) was measured by a valid weight scale (TBF-300A Body Composition Analyzer; TANITA, Arlington Heights, IL, USA). Each measurement was taken twice without delay in between, and the average of the two measurements was used in the analyses [49,50].

#### 2.2.3. Body Mass Index (BMI)

Self-reported BMI (based on self-reported height and weight) and measured BMI (based on objectively measured height and weight) were calculated as weight in kilograms divided by the square of height in meters for all participants. Both self-reported BMI and measured BMI were categorized to BMI groups based on the Western Pacific Region (WPRO) of WHO criteria pertaining to obesity [25], which categorizes people into five groups: underweight (<18.5 kg/m^2^), normal weight (18.5–22.9 kg/m^2^), overweight (23–24.9 kg/m^2^), obese stage I (25–29.9 kg/m^2^), and obese stage II (≥30 kg/m^2^).

### 2.3. Analyses Plan

Statistical analyses were conducted using STATA version 14 (College Station, Texas, TX, USA). The percentage of missing data was less than 3%. Missing data were handled using pairwise deletion. Descriptive statistics were used to characterize female Filipina and female Indonesian participants, respectively. Wilcoxon matched-pairs signed-ranks test was used to assess the reporting discrepancy of height, weight, and BMI. Mann-Whitney U test was used to assess the difference between female Filipina and female Indonesian participants regarding the reporting discrepancy of BMI. Kappa statistics and corresponding 95% confidence interval were calculated to characterize the agreement between BMI categories from self-reported and from measured BMI. Sensitivity, specificity, positive predictive value, and negative predictive value were calculated to evaluate the validity of self-reported BMI on identifying overweight or obesity. The receiver operator characteristic (ROC) curve for identifying overweight or obesity was obtained and the area under the curve (AUC) was calculated. The threshold of self-reported BMI values that maximized the percentage of individual correctly classified as being overweight or obese was determined for female Filipina and Indonesian participants, respectively.

## 3. Results

### 3.1. Participant Characteristics

Table 1 presents descriptive statistics for Filipina and Indonesian participants on age, marital status, education level, as well as self-reported and measured height, weight, BMI, and BMI categories. The age of Filipina participants ranged from 18 to 67, with a median age of 41. Most of the Filipina participants were married (44.5%) and had at least one child (78.03%). Half of the them had been working as a domestic worker in Macao for more than 39 months. Their measured height ranged from 136.0 cm to 175.0 cm (mean = 152.9, SD = 5.0), and their measured weight ranged from to 34.4 kg to 93.4 kg (mean = 57.2, SD = 8.4). Their measured BMI ranged from 16.3 kg/m^2^ to 37.5 kg/m^2^ (mean = 24.5, SD = 3.3). For Indonesian participants, their age ranged from 20 to 70, with a median age of 37. Most of them reported being married (38.0%) and roughly half had at least one child (55.71%). Half of the them had been working as a domestic worker in Macao for more than 48 months. Their measured height ranged from 139.0 cm to 165.0 cm (mean = 151.8, SD = 5.0), and their measured weight ranged from to 37.0 kg to 89.8 kg (mean = 55.8, SD = 9.91). Their measured BMI ranged from 17.0 kg/m^2^ to 36.6 kg/m^2^ (mean = 24.2, SD = 4.0).

### 3.2. Difference between Self-Reported and Measured Height and Weight

For Filipina participants, self-reported height ranged from 124.5 cm to 175.3 cm (median = 154.9, interquartile range (IQR) = 5.08), and self-reported weight ranged from 21.8 kg to 90.0 kg (median = 55.0, IQR = 10.0). The difference between self-reported and measured height (reported minus measured, same below) ranged from −19.9 cm to 17.1 cm (median = 2.6, IQR = 3.3). The difference between self-reported and measured weight ranged from −19.8 kg to 19.9 kg (median = −1.3, IQR = 3.2). The results of Wilcoxon signed-rank tests indicated that the self-reported height was larger than the measured height (z = 26.1, *p <* 0.001), and the self-reported weight was smaller than the measured weight (z = −21.3, *p <* 0.001).

For Indonesian participants, self-reported height ranged from 130.0 cm to 175.0 cm (median = 152.5, IQR = 8.0), and self-reported weight ranged from 26.3 kg to 85.0 kg (median = 53.0, IQR = 12.0). The difference between self-reported and measured height ranged from −19.0 cm to 17.5 cm (median = 1.5, IQR = 3.5). The difference between self-reported and measured weight ranged from −18.0 kg to 12.2 kg (median = −0.9 kg, IQR = 3.9). The results of Wilcoxon signed-rank test indicated that the self-reported height was larger than the measured height (z = 6.3, *p <* 0.001), and the self-reported weight was smaller than the measured weight (z = −9.5, *p <* 0.001).

### 3.3. Difference between Self-Reported and Measured BMI

For Filipina participants, self-reported BMI ranged from to 9.1 kg/m^2^ to 38.2 kg/m^2^ (median = 22.3, IQR = 4.1). The difference between self-reported and measured BMI ranged from −9.7 kg/m^2^ to 12.8 kg/m^2^ (median = −1.46, IQR = 1.7). Wilcoxon signed-rank test showed that the self-reported BMI was smaller than the measured BMI among Filipina participants (z = −27.5, *p* < 0.001).

For Indonesian participants, self-reported BMI ranged from to 13.0 to 42.8 (median = 22.6 kg/m^2^, IQR = 4.8). The difference between self-reported and measured BMI ranged from −8.3 kg/m^2^ to 9.2 kg/m^2^ (median = −0.9, IQR = 1.7). Wilcoxon signed-rank test showed that the self-reported BMI was smaller than the measured BMI (z = −9.9, *p* < 0.001) among Indonesian participants.

A Mann–Whitney U test comparing reporting discrepancy of BMI between Filipina (median = −1.46, IQR = 1.7) and Indonesian participants (median = −0.9, IQR = 1.7) found that the reporting discrepancy of BMI was greater among Filipina participants than among Indonesian participants (z = −7.1, *p <* 0.001).

### 3.4. BMI Categories

Table 2 presents BMI categories based on self-reported and measured BMI. The prevalence of overweight or obesity (BMI ≥ 23 kg/m^2^) based on self-reported BMI was 42.6% for Filipina participants and 45.9% for Indonesian participants. The prevalence of overweight or obesity based on measured BMI was 64.0% for Filipina participants and 55.1% for Indonesian participants. When comparing BMI categories based on self-reported BMI to that based on measured BMI, the kappa values indicated fair agreement for Filipina participants (kappa = 0.40, 95% confidence interval (CI): 0.34–0.40, *p* < 0.001), and moderate agreement for Indonesian participants (kappa = 0.50, 95% CI: 0.48–0.52, *p* < 0.001). The concordance of BMI categories is higher for Indonesian participants. Around 40% of the Filipina participants and 30% of the Indonesian participants were misclassified into lower BMI categories. Over 30% of the Filipina participants and 20% of the Indonesian participants who are either overweight or obese were misclassified as having a normal weight status. Compared with the gold standard of measured BMI, when self-reported BMI was used to identify overweight or obesity, the sensitivity was relatively low, while the specificity was relatively high. The sensitivity was 64.4% for Filipina participants and 78.6% for Indonesian participants. The specificity was 97.9% for Filipina participants and 93.8% for Indonesian participants. The positive predictive value was 98.2% for Filipina participants and 93.9% for Indonesian participants.

The negative predictive value was 61.2% for Filipina participants and 78.2% for Indonesian participants.

### 3.5. ROC Curve and Sensitivity and Specificity Analyses

**Figure 1:**
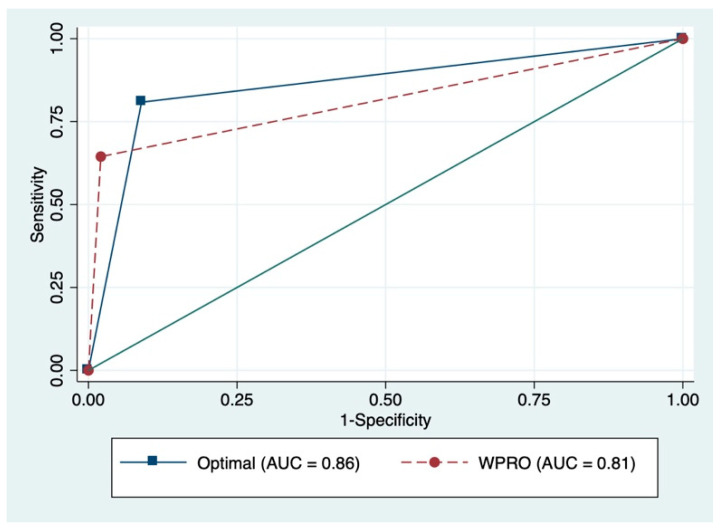
Receiver operator characteristic (ROC) curves and corresponding area under the curve (AUC) among Filipina participants. WPRO, Western Pacific Regional Office.

**Figure 2:**
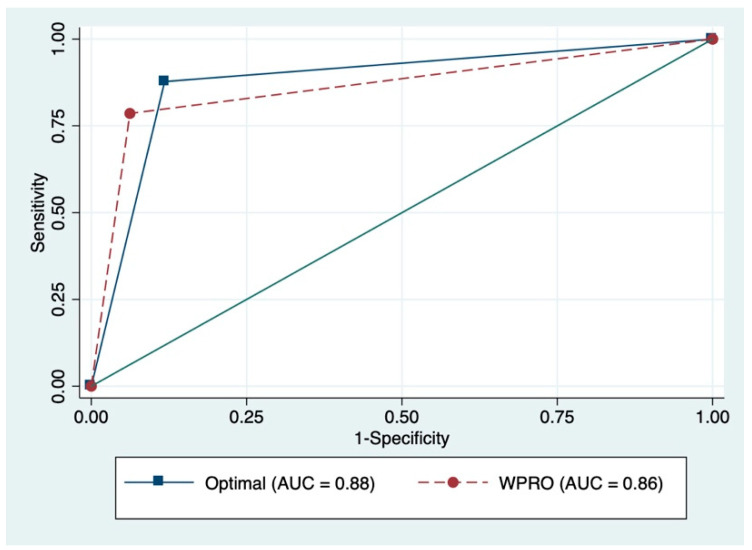
ROC curves and corresponding AUC among female Indonesian participants.

Figure 1 and Figure 2 present the ROC curves and the corresponding AUC for identifying overweight or obesity. Sensitivity and specificity analyses were conducted to identify proper cutoff points for overweight or obesity based on self-reported BMI. For Filipina participants, the ROC curve indicated an optimal cut-off point of 22.0 kg/m^2^ for BMI based on self-reported measure, which made the best compromise between sensitivity (81%) and specificity (91%) in identifying overweight or obese participants. For female Indonesian participants, the ROC curve indicated an optimal cut-off point of 22.3 kg/m^2^ for BMI based on self-reported measure, which made the best compromise between sensitivity (88%) and specificity (88%) in identifying overweight or obese participants. These new cut-off points for self-reported BMI correctly classified 84.6% of the Filipina participants and 88% of the Indonesian participants on their overweight or obesity status, which gave an estimated prevalence of overweight or obesity of 54.6% among Filipina participants and 53.5% among female Indonesian participants.

## 4. Discussion

This population representative study assessed the accuracy of self-reported height, weight, and BMI in two female migrant populations—Filipina and Indonesian domestic workers in Macao (SAR), China. A large proportion of Filipina and Indonesian participants in this study were either overweight or obese. We observed the same pattern of reporting error on height and weight in Filipina and female Indonesian participants. Height was overreported and weight was underreported. The reporting discrepancy of height and weight further results in an underestimation of BMI based on self-reported measures in both Filipina and Indonesian participants. Compared with Indonesian participants, Filipina participants have less accurate self-reported BMI. This is not surprising given that Filipina participants also had a higher prevalence of overweight or obesity than Indonesian participants. Previous studies conducted among East Asian and White non-migrant populations found that overweight or obese people were more likely to underestimate their weight [51] and their BMI [32,52]. The high prevalence of overweight and obesity in Filipina participants may thus contribute to the low accuracy of their self-reported BMI.

Although the pattern of reporting discrepancy we observed in this study is similar to that in most of the previous studies [31], it is worth noting that the magnitude of reporting discrepancy in this study, particularly among Filipina participants, is larger than that observed in other female East Asian [51,53], Southeast Asian [54], and White [55,56] non-migrant populations. Previous studies on the accuracy of self-reported measures have been conducted primarily among Western populations in high-income countries [31], which commonly attributed the reporting discrepancy to social desirability associated with ideal body image and thinness for females. However, compared with Whites, Southeast Asians preferred a higher body weight for females [37]. Southeast Asian females also displayed less body dissatisfaction than White females [37]. Social desirability may not explain the reporting discrepancy in this study among Southeast Asian female migrant workers. Given the limited access to health information and the healthcare system commonly experienced by migrant populations [20,21,22], we believe the reporting discrepancy in this study reflects a lack of health awareness and a misperception of actual weight status among Filipina and female Indonesian migrant domestic workers. This highlights the need for overweight and obesity surveillance and intervention programs for these two populations in the future.

The underestimation of BMI based on self-reported measures leads to misclassification of weight status and misidentification of overweight or obesity in both Filipina and Indonesian participants. This is consistent with the pattern observed in previous literature for East Asian and White non-migrant populations [51,52,57]. Although there was a general concordance between BMI categories from self-reported and from measured BMI, we observed a low sensitivity when self-reported BMI was used to identify overweight or obesity. The misclassification is more severe among Filipina participants. Therefore, the use of self-reported measures would lead to a substantial underestimate of overweight or obesity prevalence in these two populations. As overweight and obesity are associated with a large burden of diseases and have substantial impacts on public health, an underestimate of prevalence would mislead policymakers and limit the effectiveness of health resource allocation. Our study contributes to the existing literature by providing new cut-off points for self-reported measures to identify overweight or obesity in Filipina and female Indonesian migrant workers. The new cut-off points are 22.0 kg/m^2^ and 22.3 kg/m^2^ for Filipina and female Indonesian migrant domestic workers, respectively. With the best compromise between sensitivity and specificity, the new cut-off points can be used in future studies to identify overweight or obesity in these two populations when self-report is the only feasible way to obtain BMI.

This study has several strengths. As an effective method for reaching “hidden” and hard to reach populations, respondent-driven sampling was used to ensure population representativeness of the sample. This study is the first to document the validity of self-reported height and weight in a large population representing over 10% of the target population of vulnerable female migrant workers at the time of the study. Second, the self-reported measures and the objective measures were obtained on the same day, which minimized measurement bias introduced by potential weight change during measurement lags. Despite these strengths, several limitations of the study should be mentioned. First, as the study included only female participants, the results are not generalizable to men. Only Filipina and female Indonesian migrant domestic workers were included in this study, so the results are not generalizable to migrant populations of other ethnicities. Second, we did not have data on pre-migration weight status, so an evaluation of change in weight status and reporting discrepancy after migration is not possible. Third, we did not measure and test the mechanism of reporting discrepancy in this study. We believe that the reporting discrepancy among our study populations resulted from a lack of health awareness and a misperception of actual weight status, but this could not be directly tested in this study. Future studies may measure and test possible explanatory factors for this reporting discrepancy to have a better understanding of the actual mechanism of reporting bias.

## 5. Conclusions

BMI surveillance is crucial for obesity control, especially in high risk populations. More than half of the female migrant workers in this study were overweight or obese, which requires more surveillance efforts for BMI and implementation of evidence-based intervention programs. Although BMI derived from self-reported height and weight allows for quick and low cost obesity screening, a considerable underestimation of overweight or obesity prevalence was observed in both populations of this study. This suggests that BMI derived from self-reported height and weight is associated with substantial information bias, especially for populations with a high prevalence of overweight and obesity. When self-reported measures were used, our study provided optimal overweight or obesity cut-off points of 22.0 kg/m^2^ and 22.3 kg/m^2^ for Filipina and female Indonesian migrant domestic workers in Macao, respectively. With the best compromise between sensitivity and specificity, the new cut-off points can be used in future studies to identify overweight or obesity in these two populations using self-reported height and weight. When self-report is the only feasible way to obtain BMI, other bias correction methods, such as adjusting for other self-reported variables that are predictive for reporting discrepancy [58,59,60], should also be considered and applied to ensure a more accurate prevalence estimate for population at a high risk of overweight or obesity in future studies.

## Figures and Tables

**Table 1:** Participant characteristics.

Characteristics	Filipina (*n* = 1388)		Indonesian (*n* = 369)	
	N	%	N	%
**Age**				
18–24	28	2.0	8	2.2
25–34	298	21.5	118	32.4
35–44	555	40.0	172	47.3
45–54	414	29.8	61	16.8
55–64	90	6.5	4	1.1
65–74	3	0.2	1	0.3
**Marital Status**				
Single, never married	353	25.4	72	19.6
Married	617	44.5	140	38.0
Partnered, but not married	99	7.1	16	4.4
Separated	216	15.6	27	7.3
Legally separated	9	0.7	46	12.5
Widowed	94	6.8	67	18.2
**BMI categories (self-reported)**				
Underweight	77	5.8	25	7.0
Normal	696	52.5	168	47.1
Overweight	258	19.5	65	18.2
Obese I	256	19.3	72	20.2
Obese II	38	2.9	27	7.6
**BMI categories (measured)**				
Underweight	23	1.7	14	3.8
Normal	468	34.3	150	41.1
Overweight	341	25.0	70	19.2
Obese I	455	33.4	100	27.4
Obese II	76	5.6	31	8.5

BMI: body mass index.

**Table 2:** BMI categories according to self-reported and measured BMI.

Self-Reported BMI (kg/m^2^)						
Measured BMI (kg/m^2^)	Underweight	Normal	Overweight	Obese Stage I	Obese Stage II	Kappa
**Filipina (*n* = 1388)**						
Underweight	18	4	0	0	0	0.4 *p* < 0.001
Normal	49	400	8	2	0	
Overweight	6	222	94	10	0	
Obese stage I	1	69	149	213	6	
Obese stage II	0	1	7	31	32	
**Indonesian (*n* = 369)**						
Underweight	7	6	1	0	0	0.5 *p* < 0.001
Normal	16	122	4	5	0	
Overweight	2	31	28	7	1	
Obese stage I	0	8	32	53	4	
Obese stage II	0	1	0	7	22	

BMI: body mass index.
